# Remnant of a non-patent ductus arteriosus mimicking traumatic thoracic aorta transection: a case report

**DOI:** 10.1186/1749-8090-5-24

**Published:** 2010-04-09

**Authors:** Efstratios E Apostolakis, Nikolaos G Baikoussis, Christina Kalogeropoulou, Efstratios Koletsis, Ioanna Koniari, Dimitrios Karnabatidis, Menelaos Karanikolas

**Affiliations:** 1 Cardiothoracic Surgery Department. University Hospital, Patras School of Medicine. Patras, Greece; 2 Department of Interventional Radiology. University Hospital, Patras School of Medicine. Patras, Greece; 3 Department of Anaesthesiology and Critical Care Medicine. University Hospital of Patras. Patras, Greece

## Abstract

We present an interesting case of a 53-year-old man with a non-patent ductus arteriosus erroneously diagnosed as acute thoracic aorta transection after a car accident. The aortography revealed a "rupture" of the linear inner curve of the aorta in the isthmus area, as well as a protrusion of the aortic lumen in the corresponding area. During the followed thoracotomy an intact thoracic aorta and the remnant of a non-patent ligamentum arteriosum were found. It is the first reported case and we review all the possible entities which may give a false-positive image of traumatic aortic transection.

## Background

Aortography was for many years the "gold standard" in diagnosis of acute traumatic aortic rupture against the two other methods of diagnostic imaging: CT-angiography and transesophageal ECHO [1]. Its sensitivity and specificity in experienced hands approaches 100% [2]. However, in rare cases a false-positive or false-negative imaging may be observed. For the false positive images of traumatic rupture the most common causes are local atherosclerotic lesions of the aortic wall, ductal diverticula [3], remnant of non-patent ductus arteriosus or pre-existent aneurysm of the isthmus area [4]. We describe herein a case of an injured patient with high-suspicion index of traumatic aortic rupture, which was based on a false-positive aortography.

## Case presentation

**Figure 1:**
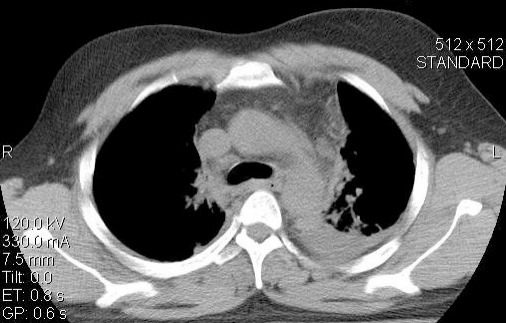
**Thorax-CT of the patient indicating left hemothorax, left lung contusion in its posterior segments and a diffusing periaortic hematoma in the aortic isthmus area**.

**Figure 2:**
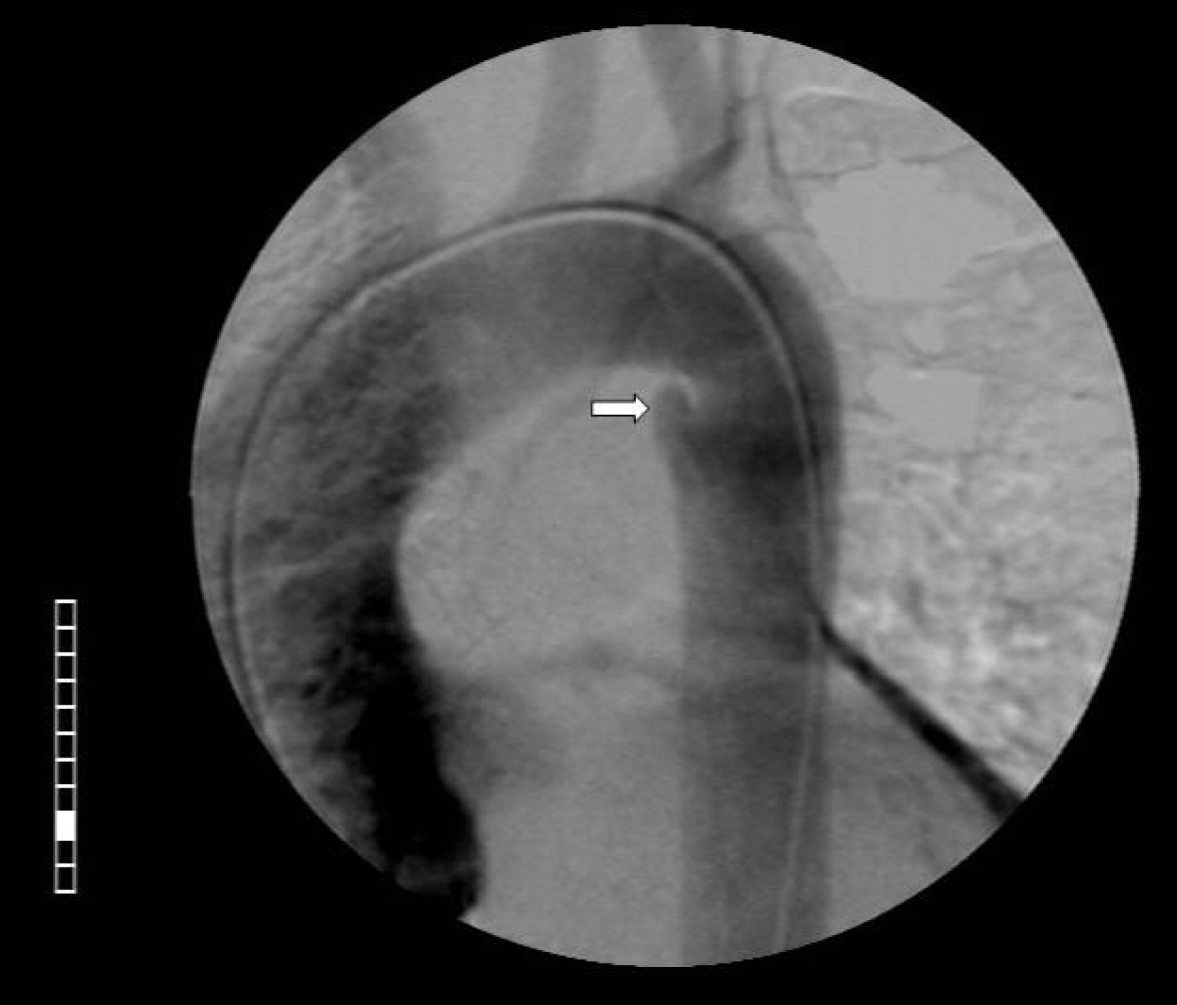
**Aortography showed an interruption (the so called "linear tear") of the normal contour of the thoracic aorta in the corresponding area**. A protrusion of the aortic lumen in the inner curve of the aorta is indicating the disruption of the intima and beginning of a pseudoaneurysm. The preoperative evaluation of imaging was: "findings indicating a traumatic rupture of aortic isthmus".

A 53 year-old man was transported from another hospital with the high suspicion of a traumatic aortic rupture after acute blunt thoracic trauma. Following a high speed car accident he was admitted in another hospital with injuries in the chest and fracture of the left femur. A thorax-CT scan was performed without contrast medium because of a known chronic renal failure (creatinine levels = 2.2 mMol/L). It showed hemothorax on the left, minimal left lung contusions (of the posterior segments), rib fractures and a periaortic hematoma at the level of the isthmus area (figure 1). Because of a high-suspicion index of thoracic aortic rupture, we decided to do an emergency aortography. It revealed an interruption of the normal contour of the thoracic aorta in the aortic isthmus area. A protrusion of the aortic lumen in the corresponding inner curve of the aorta supported our suspicion for the disruption of the intima and the initiation of a pseudoaneurysm's process (figure 2). Therefore, an emergency operation (the interventional management was abandoned because of technical reasons) by using partial right femoro-femoral bypass for aortic isthmus repair was decided. Surprisingly, and after a postero-lateral thoracotomy at the 4^th ^intercostals space, we inspected an "intact" outer thoracic aortic wall, without haematoma or related pathology at the aortic isthmus area. However, because we did not totally exclude a possible limited disruption of the intima, or even another pathology (see discussion), we decided to check from inside the thoracic aorta. Following proximal and distal dissection of the aorta, a partial cardiopulmonary bypass was initiated with flow level 2-2.6 L/min to restore a distal aortic pressure of >55-60 mm. After double clamping and vertical opening of the aorta wall, an intact endothelium was observed. In the inner curve of the aortic isthmus area and in the site of occluded ligamentum arteriosum, a local vestigial dilatation 0.5 × 0.8 cm with normal endothelium lining was observed. Two stitches of prolene 4-0 reinforced with Teflon felt was used to obliterate this remnant. The aortotomy was then closed, the cardiopulmonary bypass was interrupted and the rest of operation was as usually. The patient was extubated after 8 hours and his postoperative course was uneventful. The patient underwent successfully on the 9^th ^postoperative day the surgical management of his right femur fracture and was discharged from the hospital on the 17^th ^postoperative day in good condition.

## Conclusions

In every case of suspicion of traumatic aortic transection, the imaging diagnosis is based on spiral CT-angiography or transesophageal echocardiography (TEE), and rarely on the conventional aortography. Aortography is considered as the exam with the higher specificity and sensibility approaching the 100% [2]. However, rare preexistent pathological conditions may obscure the clearness of these imaging examinations. Indeed, these conditions may mimic an aortic rupture and in this way give false-positive results. Therefore, it should be taken under consideration by the operator of the angio-CT, or of the TEE, to avoid any pitfall for the final diagnosis. The four rare entities which may give false-positive imaging of aortic rupture in the region of the isthmus are the following. **A**. Remnant of a non-patent ductus arteriosus. This vestigial may appear as a local protrusion of the aortic extremity of the ductus-as in our case- or as a scarry remnant which on the CT angiography creates a transformation and an angulation with compression between aorta and pulmonary artery (scarry remnant forming the "corner point" of a compression between aorta and pulmonary artery) [5]. On this remnant of the ductus arteriosus may be developed later in the adult life, infective endocarditis [6].

**B**. Aneurysm of a non-patent ductus arteriosus. They usually arise from the aortic extreme of the ductus and may compress the nearest organs like trachea and esophagus, giving related symptoms [4,5]. **C**. Aortic diverticulum. It is commonly thought to be a remnant of the closed ligamentum or ductus arteriosus. However some authors support the hypothesis that it is a remnant of the right dorsal aortic root [7]. It is described in thoracic aortography as a large bulge on the lesser curvature of the aortic isthmus, in patients with a left aortic arch and normal origin of the brachiocephalic arteries.

**D**. Calcification of the ligamentum arteriosum and/or of the aortic wall in the aortic isthmus area. This calcification in the adults may be in several patterns such as curvilinear, punctate or clumped, and in incidence up to 65% [8]. In our case, we chose the surgical instead of the endovascular-intervention, for the following two reasons. First, because an endovascular graft was not in time available, and second, there were no contraindications for surgical intervention (brain injury, coagulation's abnormalities, etc). Despite of absence of signs of aortic transection during the inspection of the thoracic aorta (intramural hematoma, periaortic infiltration, etc), the image of aortography posed us in a dilemma, taken in consideration our experience and the bibliographic data; there is not traumatic aortic rupture without haematic infiltration. According these data, we decided open the aorta to elucidate the differential diagnosis about the given image of aortography.

## Competing interests

The authors declare that they have no competing interests.

## Authors' contributions

All authors: 1. have made substantial contributions to conception and design, or acquisition of data, or analysis and interpretation of data; 2. have been involved in drafting the manuscript or revisiting it critically for important intellectual content; 3. have given final approval of the version to be published.

## Consent

Written informed consent was obtained from the patient for publication of this case report and accompanying images. A copy of the written consent is available for review by the Editor-in-Chief of this journal.
